# Caregiving activities causing occupational low back pain in Japanese social welfare facilities and hospitals

**DOI:** 10.1371/journal.pone.0342979

**Published:** 2026-02-13

**Authors:** Kazuyuki Iwakiri, Keiichi Miki, Takeshi Sasaki

**Affiliations:** National Institute of Occupational Safety and Health, Japan; Iran University of Medical Sciences, IRAN, ISLAMIC REPUBLIC OF

## Abstract

**Background:**

Occupational low back pain (LBP) has increased in Japanese social welfare facilities and hospitals. Understanding its occurrence is the first step in addressing this issue.

**Aim:**

This study investigated the caregiving activities that cause occupational LBP incidents in these contexts.

**Methods:**

The study analyzed 2,722 incidents of occupational LBP among staff in social welfare facilities and hospitals resulting in four or more days absent from work. Data were extracted from accident occurrences and causes in the 2018–2019 *Reports of Worker Casualties*. The caregiving situations related to the occupational LBP incidents at each facility were then analyzed.

**Results:**

Approximately half of the occupational LBP incidents surveyed occurred during transfer assistance. This assistance was mainly associated with eating, bathing, and toileting in both facilities and frequently occurred during patient transfers between a bed and a wheelchair. In social welfare facilities, nontransfer assistance also contributed significantly, which included bathing, toileting, childcare, diaper changing, lying, standing, sitting, walking, and car transportation. In hospitals, nontransfer assistance such as providing support for lying, diaper changing, medical care, and sitting were occupational LBP risk factors. Furthermore, most incidents of occupational LBP occurred among staff who worked alone during day shifts.

**Conclusion:**

Caregiving activities involving transfer assistance, such as bathing, toileting, and eating, were common risk factors in social welfare facilities and hospitals. However, the specific nontransfer assistance activities contributing to occupational LBP varied by facility. To effectively reduce the incidence of occupational LBP, prevention strategies should focus on these high-risk activities according to facility type and the types of caregiving required.

## Introduction

Occupational low back pain (LBP), which is recognized and compensated as a workplace accident in Japan, accounts for approximately 60% of all occupational injuries and has been increasing in recent years [1]. In the healthcare and welfare services industry, occupational LBP is especially prevalent, where its increasing incidence contributes to a broader rise in overall occupational LBP cases [2].

The healthcare and welfare services industry includes social welfare facilities and hospitals. Social welfare facilities offer welfare-related services to older people, children, and people experiencing disability, while hospitals focus on providing healthcare to patients. Caregivers comprise the bulk of the workforce in the former, whereas nurses comprise a significant portion of the workforce in the latter. In Japan, the job content and qualifications of caregivers and nurses are separate. Japan’s aging population is expected to grow further in the future, and the demand for these positions is also expected to increase [3,4]. As these employees increase in number, incidents of occupational LBP will rise accordingly. Therefore, measures to prevent occupational LBP in social welfare facilities and hospitals must be urgently considered.

The first step in addressing this issue is to understand the occurrence of occupational LBP. Recent surveys of specific facilities have reported significant back strain for caregivers and nurses when assisting with transfers, bathing, toileting, and diaper changing [5–10]. However, no recent studies have examined the occurrences and causes of occupational LBP incidents that are officially recognized as workplace accidents.

When a worker suffers a workplace accident, the employer must submit a Report of Worker Casualties to the Ministry of Health, Labour and Welfare. These reports are classified as less than or more than four days of absence from work. Reports for less than four days of absence are simple and given within a summary report for all incidents from the past three months. However, reports on incidents causing more than four days of absence must be detailed and reported every time a workplace accident occurs because it is considered a vital disease. This report must describe the incident’s occurrence and causes in free text and illustrate the situation at the time of the accident. Analyzing these descriptions and illustrations makes the reality of occupational LBP in social welfare facilities and hospitals apparent.

This study investigated the relationship between occupational LBP and caregiving activities in social welfare facilities and hospitals using workplace accident data for incidents that caused more than four days of absence. As a result, it identified the caregiving activities that should be prioritized to improve occupational LBP rates.

## Materials and methods

### Research design

The study surveyed all cases of occupational LBP causing four or more absent days from work as certified by the Japanese Ministry of Health, Labour and Welfare from 2018 to 2019. The target data for the analysis were created by removing duplicate cases, cases related to work other than caregiving and nursing, cases that occurred outside social welfare facilities or hospitals, and cases occurring during unknown care work.

In social welfare facilities and hospitals, transfers from bed to wheelchair and from wheelchair to stretcher are performed in various situations, such as eating, bathing, and toileting. Transfer assistance is a significant risk factor for occupational LBP in caregiving and nursing [5–7,9–11]. Understanding the caregiving situation for transfer assistance is necessary to clarify the occurrence of occupational LBP. Therefore, the data were divided into transfer assistance and nontransfer assistance. Nontransfer assistance was specific to the caregiving context: for example, washing the body of a person requiring care or a patient while bathing or wiping the buttocks while toileting. The target data were divided into four groups: (A) cases that occurred during transfer assistance in social welfare facilities; (B) cases that occurred during nontransfer assistance in social welfare facilities; (C) cases that occurred during transfer assistance in hospitals; and (D) cases that occurred during nontransfer assistance in hospitals. These four categories were referred to as facility assistance types. Because the data distributions in 2018 and 2019 were not significantly different, the data for the two years were compiled.

Data were collected from February 2, 2024, to March 31, 2025. The authors accessed personally identifiable information during this period, but this access was strictly confined to a secure, isolated room and a dedicated computer that was not connected to any external networks. As the data comprised historical records of workplace accidents, it was not considered feasible to contact all the individuals involved. Therefore, in accordance with the principles of the Declaration of Helsinki, the study employed an opt-out approach, allowing participants to decline participation and publication of the study without providing a reason. Ethical approval was obtained from the Ethics Board of the National Institute of Occupational Safety and Health (Registration ID: 2021N07, 2023N09).

### Reports of worker casualties

Gender, age, years of experience (i.e., the number of years employed at the given workplace), duration of absence from work, the day and time of the accident, the number of workers involved in the accident, and the occurrence and cause of occupational LBP (including a description and an illustration) were extracted from the *Reports of Worker Casualties* for incidents causing four or more days of absence. The duration of work absence was considered an indicator of the severity of occupational LBP. The descriptions and illustrations concerning the occurrence and cause of occupational LBP were digitized, and the caregiving situations, assistance types, and origin and destination of transfers were extracted. The number of occupational LBP cases that occurred during the use of transfer-assistive devices—including lifts, sliding sheets, and sliding boards—was also extracted.

Caregiving situations were categorized into both transfer and nontransfer assistance with eating, bathing, toileting, diaper changing, lying, sitting, standing, walking, car transportation, medical support, childcare, and other situations. Here, lying situations primarily involved adjusting a bed’s position and changing the posture of care recipients or patients, while sitting situations involved adjusting their sitting position to ensure proper posture. Standing situations mainly involved assisting care recipients or patients in standing up, and walking situations included physically supporting them to prevent falls while moving. Car transportation included boarding and alighting assistance for care recipients and patients into a shuttle car and helping them into their seats, while medical support encompassed all medical procedures and daily life support. Childcare included holding and carrying infants and young children in daycare and pediatric settings, primarily undertaken by nursery workers. Other situations included those where the caregiving context was either unspecified or did not fall into the abovementioned categories, such as the caregivers’ and nurses’ independent movements, including during cleaning, recreation, and communication. For transfer assistance cases, the origins and destinations of the transfer were categorized as bed, wheelchair, bathtub, floor, toilet, chair, stretcher, car, and other. Here, the chair category included shower chairs and shower carriers.

### Data analysis

A chi-squared (χ2) test was performed to compare the number of occupational LBP incidents among groups A, B, C, and D. When significant differences were found, a residual analysis was conducted to compare those differences between the groups. Moreover, the duration of work absence across groups and caregiving situations was compared using the Kruskal–Wallis or Mann–Whitney U tests. When significant differences were identified, the Dunn–Bonferroni test was performed for pairwise comparisons between two conditions. The residual analysis considered adjusted residuals of ≥1.96 and ≤−1.96 significant at a risk level of <5%. A correspondence analysis was performed to analyze the relationship between caregiving situations and the type of facility assistance. A hierarchical cluster analysis was performed using x and y coordinates derived from the correspondence analysis as variables to categorize the caregiving situations associated with each group. IBM SPSS version 27 was used for the statistical analysis, with a significance level of <5%.

## Results

### Survey data

**Fig 1:**
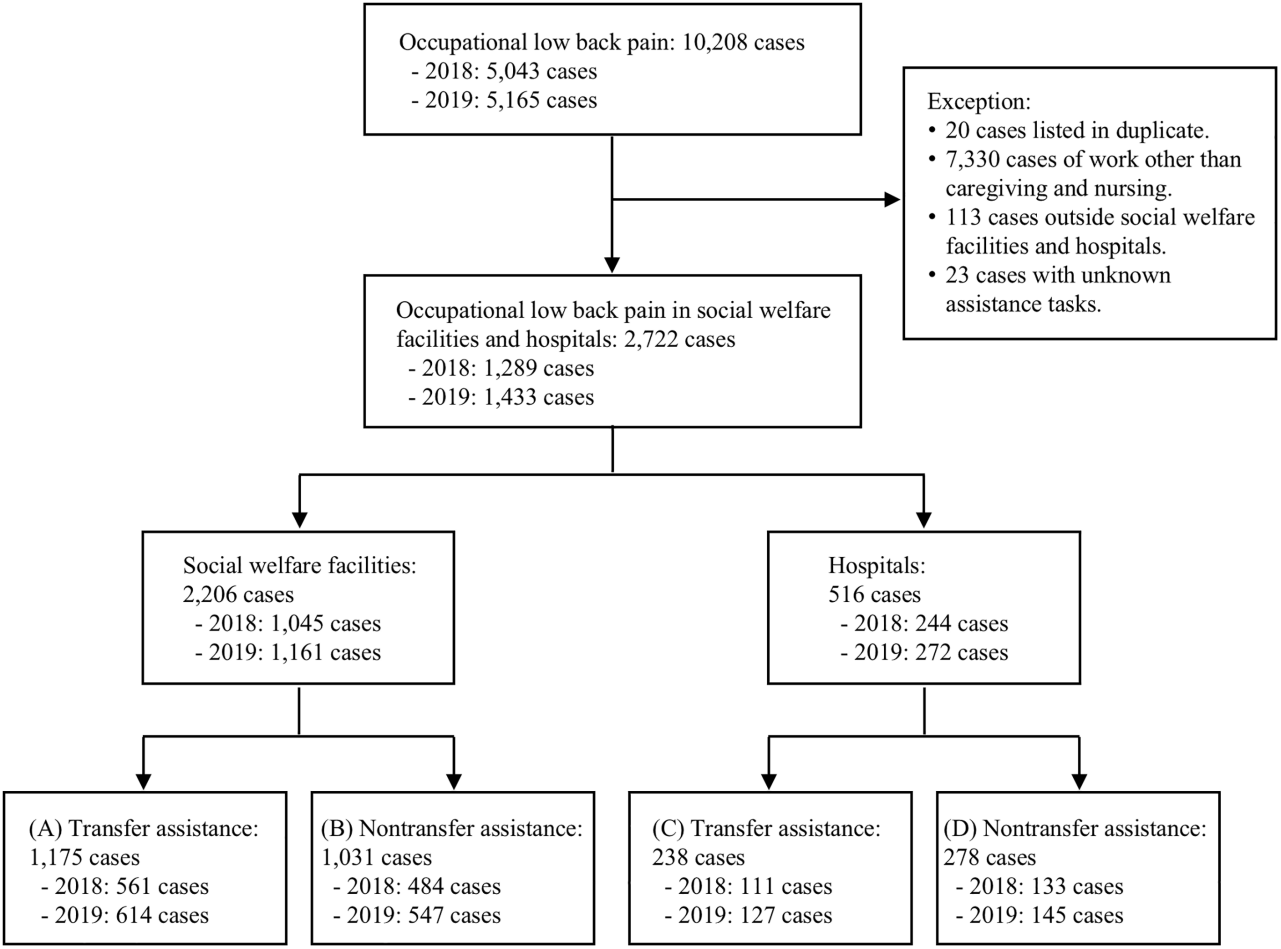
Data survey and organization process.

The initial number of all occupational LBP incidents in 2018–2019 causing four or more days of absence from work, according to the Japanese Ministry of Health, Labour, and Welfare, was 10,208. Of these, 10,148 cases (99.4%) were for acute pain and 60 cases (0.6%) were for chronic pain. The following cases were then removed from the data pool: 20 duplicate cases, 7,330 cases related to work outside caregiving and nursing, 113 cases that occurred outside social welfare facilities and hospitals, and 23 cases that occurred during unknown care work. The final target data comprised 2,722 cases, which occurred among workers in social welfare facilities and hospitals who were engaged in caregiving and nursing (Fig 1).

The staff at the social welfare facilities primarily comprised caregivers, along with some nurses and childcare workers. Hospital staff mainly included nurses, some caregivers, and some childcare workers, but not physicians. Childcare workers in the facilities were employed in daycare centers, whereas those in hospitals worked in pediatric departments and nursery rooms. The grouping of the 2,722 cases into facility assistance types resulted in (A) 1,175 social welfare facilities transfer assistance cases; (B) 1,031 social welfare facilities nontransfer assistance cases; (C) 238 hospital transfer assistance cases; and (D) 278 hospital nontransfer assistance cases (Table 1).

**Table 1:** Workers’ demographic information.

*n* (%)	Social welfare facilities (*n* = 2,206)		Hospitals (*n* = 516)		*P*-value^a^
	(A) Transfer assistance (*n* = 1,175)	(B) Nontransfer assistance (*n* = 1,031)	(C) Transfer assistance (*n* = 238)	(D) Nontransfer assistance (*n* = 278)	
**Sex**					0.104
Male	312 (26.6)	246 (23.9)	74 (31.1)	68 (24.5)	
Female	863 (73.4)	785 (76.1)	164 (68.9)	210 (75.5)	
**Age (years)**					0.002
<30	237 (20.2)	189 (18.3)	53 (22.3)	59 (21.2)	
30–39	262 (22.3)^#^	251 (24.3)	69 (29.0)	76 (27.3)	
40–49	289 (24.6)	281 (27.3)	54 (22.7)	78 (28.1)	
50–59	230 (19.6)	199 (19.3)	42 (17.6)	54 (19.4)	
≥60	157 (13.4)^*^	111 (10.8)	20 (8.4)	11 (4.0)^#^	
**Experience (years)**					<0.001
<1	310 (26.4)^*^	237 (23.0)	45 (18.9)	40 (14.4)^#^	
1–2	258 (22.0)	247 (24.0)^*^	45 (18.9)	45 (16.2)^#^	
3–4	136 (11.6)	149 (14.5)	28 (11.8)	37 (13.3)	
5–9	269 (22.9)	201 (19.5)	45 (18.9)	61 (21.9)	
≥10	202 (17.2)^#^	197 (19.1)	75 (31.5)^*^	95 (34.2)^*^	
**Absence from work (days)**					0.443
4–7 days	299 (25.4)	303 (29.4)	69 (29.0)	72 (25.9)	
8–14 days	335 (28.5)	303 (29.4)	75 (31.5)	79 (28.4)	
15–30 days	303 (25.8)	247 (24.0)	54 (22.7)	71 (25.5)	
≥31 days	238 (20.3)	178 (17.3)	40 (16.8)	56 (20.1)	

a : The chi-squared (*χ2*) tests were performed to compare groups A–D.

* : Adjusted standardized residuals in the residual analysis of the chi-square test were ≥1.96 (*p* < 0.05).

# : Adjusted standardized residuals in the residual analysis of the chi-square test were ≤−1.96 (*p* < 0.05).

### Demographic data of workers

The proportion of female workers was approximately 70%–75%. The mean ages and SD were 43.0 ± 13.6 years in Group A, 42.6 ± 12.9 years in Group B, 40.6 ± 12.7 years in Group C, and 40.2 ± 11.4 years in Group D. Compared with workers in hospitals, few workers in social welfare facilities were in their 30s; most were in their 60s or older. The years of experience were 4.8 ± 5.3 years for Group A, 5.3 ± 6.6 years for Group B, 8.3 ± 9.7 years for Group C, and 8.5 ± 8.9 years for Group D. Hospital workers were less likely to have <2 years of experience and more likely to have >10 years of experience compared with social welfare facility workers. The days absent from work were 26.8 ± 31.5 days in Group A, 24.0 ± 28.7 days in Group B, 22.9 ± 28.2 days in Group C, and 26.7 ± 30.5 days in Group D. There were no significant differences between the groups in gender or days absent from work.

### Caregiving situations and facility assistance types

Occupational LBP due to transfer assistance accounted for 51.9% of all cases, with 53.3% in social welfare facilities, and 46.1% in hospitals (Table 2). The chi-square test results showed significant differences in the distribution of occupational LBP concerning caregiving situations and facility assistance types. Residual analysis showed that the proportion of occupational LBP incidents associated with eating was higher in Group A than in other groups. The proportion of occupational LBP incidents related to bathing was lowest in Group D. For toileting, standing, walking, car transportation, and childcare, the proportion of occupational LBP incidents was higher in Group B than in the other groups. The proportion of occupational LBP incidents associated with diaper changing, lying, and sitting was higher in Groups B and D than A or C. The proportion of occupational LBP incidents related to medical support was higher in Group D than other groups. The proportion of occupational LBP incidents in other situations was higher in Groups A and C than B and D.

**Table 2:** Occupational low back pain incidents by situation and facility.

Number of cases (%)	Caregiving situations								
	Eating	Bathing	Toileting	Diaper changing			Lying	Sitting	Standing
**Social welfare facilities**									
**(A) Transfer**	57* (55.3)	213(43.6)	149(44.3)	16#(7.8)			0#(0.0)	0#(0.0)	0#(0.0)
**(B) Nontransfer**	28#(27.2)	195(40.0)	153*(45.5)	125*(60.7)			101*(58.7)	30*(78.9)	60*(87.0)
**Hospitals**									
**(C) Transfer**	8(7.8)	49(10.0)	20(6.0)	5#(2.4)			0#(0.0)	0(0.0)	0#(0.0)
**(D) Nontransfer**	10(9.7)	31#(6.4)	14#(4.2)	60*(29.1)			71*(41.3)	8*(21.1)	9(13.0)
**Total**	103(100)	488(100)	336(100)	206(100)			172(100)	38(100)	69(100)
**Number of cases (%)**	**Caregiving situations (cont.)**								**Total**
	**Walking**		**Car transportation**		**Medical support**	**Childcare**		**Other**	
**Social welfare facilities**									
**(A) Transfer**	0#(0.0)		14(33.3)		3#(2.3)	0#(0.0)		723*(75.2)	1,175(43.2)
**(B) Nontransfer**	25*(96.2)		25*(59.5)		59(45.0)	147*(98.0)		83#(8.6)	1,031(37.9)
**Hospitals**									
**(C) Transfer**	0(0.0)		0#(0.0)		16(12.2)	0#(0.0)		140*(14.6)	238(8.7)
**(D) Nontransfer**	1(3.8)		3(7.1)		53*(40.5)	3#(2.0)		15#(1.6)	278(10.2)
**Total**	26(100)		42(100)		131(100)	150(100)		961(100)	2,722(100)

* : Adjusted standardized residuals in the residual analysis of the chi-square test were ≥1.96 (*p* < 0.05).

# : Adjusted standardized residuals in the residual analysis of the chi-square test were ≤−1.96 (*p* < 0.05).

Table 3 presents the durations of work absence for occupational LBP incidents. Work absence is associated with the severity of occupational LBP, with longer durations indicating more severe conditions. The results of the Kruskal–Wallis or Mann–Whitney U tests showed that Group A exhibited longer work absence due to occupational LBP associated with eating and other situations than Group B. Regarding occupational LBP due to diaper changing, Group D displayed longer work absence than Group B. Meanwhile, for childcare-related occupational LBP, Group B reported longer work absence than Group D. In addition, within-group comparisons revealed that in Group B, work absence due to occupational LBP associated with eating was significantly shorter than that associated with toileting, standing, and other situations. However, no significant differences were observed in the other categories.

**Table 3:** Work absence by situation and facility.

Number of cases (%)	Caregiving situations						
	Eating	Bathing	Toileting	Diaper changing	Lying	Sitting	Standing
**Social welfare facilities**							
**(A) Transfer**	21.5 ± 21.0^*1^	23.5 ± 25.5	27.0 ± 29.6	26.4 ± 29.0	–	–	–
**(B) Nontransfer**	12.9 ± 16.4^#^	20.3 ± 26.5	26.5 ± 26.0	21.7 ± 24.0	27.8 ± 36.4	28.5 ± 33.6	30.8 ± 37.5
**Hospitals**							
**(C) Transfer**	23.9 ± 25.6	21.3 ± 18.9	23.3 ± 28.3	29.4 ± 35.2	–	–	–
**(D) Nontransfer**	15.6 ± 12.1	21.6 ± 24.5	47.9 ± 53.5	30.2 ± 31.2^*2^	28.4 ± 32.0	19.5 ± 17.8	31.6 ± 45.5
**Total**	18.8 ± 19.7	21.9 ± 25.2	27.4 ± 29.5	24.8 ± 27.0	28.1 ± 34.5	26.6 ± 31.0	30.9 ± 38.3
**Number of cases (%)**	**Caregiving situations (cont.)**						**Total**
	**Walking**	**Car transportation**	**Medical support**		**Childcare**	**Other**	
**Social welfare facilities**							
**(A) Transfer**	–	32.9 ± 37.8	11.7 ± 4.0		–	28.2 ± 34.0^*1^	26.8 ± 31.5
**(B) Nontransfer**	22.6 ± 25.2	31.0 ± 34.5	24.4 ± 29.0		22.0 ± 29.2^*3^	25.3 ± 25.3	24.0 ± 28.7
**Hospitals**							
**(C) Transfer**	–	–	20.8 ± 16.9		–	23.4 ± 31.9	22.9 ± 28.2
**(D) Nontransfer**	90.0 ± 0.0	20.0 ± 20.0	21.2 ± 24.0		5.0 ± 1.7	23.7 ± 20.4	26.7 ± 30.5
**Total**	25.2 ± 28.0	30.8 ± 34.4	22.4 ± 25.4		21.7 ± 29.0	27.2 ± 32.9	25.4 ± 30.1

*1 : A > B, * ^2^: D > B, * ^3^: B > D (p < 0.05).

# : Eating < toileting, standing, or other situations (p < 0.05).

**Fig 2:**
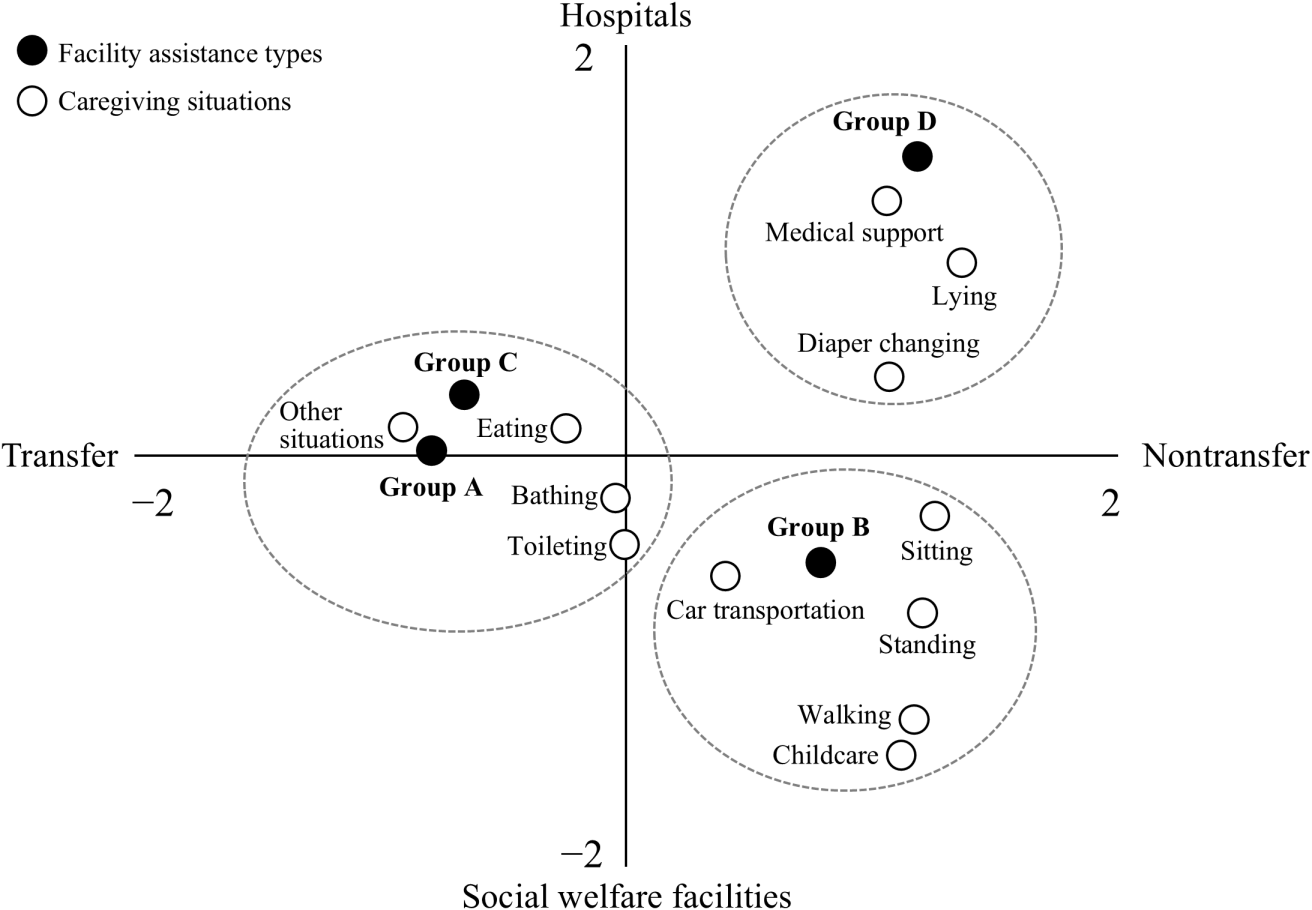
Case correspondence with caregiving situation and facility type. Group A = Transfer assistance in social welfare facilities; Group B = Nontransfer assistance in social welfare facilities; Group C = Transfer assistance in hospitals; Group D = Nontransfer assistance in hospitals. Dotted circles represent the stratified categories in the hierarchical cluster analysis.

Fig 2 shows the correspondence analysis map. The vertical axis indicates the facility type (social welfare facility or hospital), and the horizontal axis indicates assistance type (transfer or nontransfer). The dotted circles represent the three categories aggregated in the hierarchical cluster analysis. Transfer assistance in social welfare facilities and hospitals was classified into the same category, placed on the left side of the figure, and closely related to eating, bathing, toileting, and other situations (Groups A and C). Nontransfer assistance in social welfare facilities was positioned in the lower right of the figure and was closely related to sitting, standing, walking, car transportation, and childcare (Group B). Hospital nontransfer assistance was positioned in the upper right of the figure and was closely related to diaper changing, lying, and medical support (Group D).

### Transfer origin and destination

The most significant number of occupational LBP cases associated with transfer assistance in social welfare facilities occurred during bed-to-wheelchair transfers, followed by wheelchair-to-bed, toilet-to-wheelchair, floor-to-wheelchair, wheelchair-to-toilet, chair-to-wheelchair, and wheelchair-to-chair (Table 4). The highest number of occupational LBP cases associated with hospital transfer assistance occurred during bed-to-wheelchair transfers, followed by wheelchair-to-bed and bed-to-stretcher (Table 5).

**Table 4:** Cases by transfer origin and destination in social welfare facilities.

Number of cases (%)		Transfer destination									
		Bed	WCh	Bath	Floor	Toilet	Chair	Str	Car	Other	Total
**Transfer** **origin**	**Bed**		358 (33.0)	7 (0.6)		15 (1.4)	3 (0.3)	7 (0.6)	1 (0.1)	1 (0.1)	392 (36.1)
	**WCh**	277 (25.5)	8 (0.7)	4 (0.4)	9 (0.8)	41 (3.8)	32 (2.9)	24 (2.2)	1 (0.1)	6 (0.6)	402 (37.0)
	**Bath**	7 (0.6)	7 (0.6)				12 (1.1)			11 (1.0)	37 (3.4)
	**Floor**	36 (3.3)	44 (4.1)	1 (0.1)	1 (0.1)	6 (0.6)	10 (0.9)		2 (0.2)		100 (9.2)
	**Toilet**	8 (0.7)	47 (4.3)				1 (0.1)				56 (5.2)
	**Chair**	9 (0.8)	36 (3.3)	7 (0.6)	1 (0.1)	1 (0.1)	2 (0.2)			1 (0.1)	57 (5.2)
	**Str**	8 (0.7)	10 (0.9)				1 (0.1)	2 (0.2)		2 (0.2)	23 (2.1)
	**Car**	1 (0.1)	5 (0.5)								6 (0.6)
	**Other**	4 (0.4)	3 (0.3)	3 (0.3)			1 (0.1)	2 (0.2)			13 (1.2)
	**Total**	350 (32.2)	518 (47.7)	22 (2.0)	11 (1.0)	63 (5.8)	62 (5.7)	35 (3.2)	4 (0.4)	21 (1.9)	1,086 (100)

WCh = Wheelchair; Bath = Bathtub; Str = Stretcher. Blank spaces indicate “0 (0.0).”

**Table 5:** Cases by transfer origin and destination in hospitals.

Number of cases (%)		Transfer destination									
		Bed	WCh	Bath	Floor	Toilet	Chair	Str	Car	Other	Total
**Transfer** **origin**	**Bed**		81 (37.5)			5 (2.3)		17 (7.9)		1 (0.5)	104 (48.1)
	**WCh**	51 (23.6)	1 (0.5)			2 (0.9)	2 (0.9)	4 (1.9)	2 (0.9)	3 (1.4)	65 (30.1)
	**Bath**				1 (0.5)						1 (0.5)
	**Floor**	4 (1.9)	3 (1.4)				1 (0.5)	2 (0.9)	1 (0.5)		11 (5.1)
	**Toilet**	2 (0.9)	7 (3.2)								9 (4.2)
	**Chair**							2 (0.9)		1 (0.5)	3 (1.4)
	**Str**	1 (0.5)	2 (0.9)				1 (0.5)				4 (1.9)
	**Car**	7 (3.2)	2 (0.9)	1 (0.5)				4 (1.9)		2 (0.9)	16 (7.4)
	**Other**		2 (0.9)					1 (0.5)			3 (1.4)
	**Total**	65 (30.1)	98 (45.4)	1 (0.5)	1 (0.5)	7 (3.2)	4 (1.9)	30 (13.9)	3 (1.4)	7 (3.2)	216 (100)

WCh = Wheelchair; Bath = Bathtub; Str = Stretcher. Blank spaces indicate “0 (0.0).”

### Status of occupational LBP occurrences

Only six cases of occupational LBP occurred when workers used transfer-assist devices. One case occurred during the use of a lift, two during the use of a sliding sheet, and three during the use of a sliding board. The incidents were attributed to workers adopting inappropriate working postures during transfer assistance.

The results of the Chi-square test showed no significant differences in terms of the day of the week of occupational LBP incidents; however, this study observed significant differences in the distribution of occupational LBP by accident time (Table 6). Across all groups, occupational LBP occurred most frequently between 6:00 and 17:59, with the highest incidence between 9:00 and 11:59. Residual analysis results showed that, in Group A, the frequency of occurrence during 00:00–02:59 and 09:00–11:59 were lower than those in the other groups, whereas the frequency during 06:00–08:59 was higher. For Group B, the frequency during 00:00–02:59 was higher than that for the other groups, and for Group C, the frequency during 09:00–11:59 was highest. Meanwhile, for Group D, the frequency during 09:00–11:59 was higher than that for the other groups whereas the frequency during 06:00–08:59 was lower.

**Table 6:** Times when occupational low back pain accidents occurred.

Number of cases (%)	00:00 –02:59	03:00 –05:59	06:00 –08:59	09:00 –11:59	12:00 –14:59	15:00 –17:59	18:00 –20:59	21:00 –23:59	Total
**Social welfare facilities**									
**(A) Transfer**	6^#^ (19.4)	28 (40.6)	175^*^ (52.4)	456^#^ (40.0)	229 (46.5)	175 (43.4)	80 (42.8)	26 (38.8)	1,175 (43.2)
**(B) Nontransfer**	22^*^ (71.0)	27 (39.1)	117 (35.0)	430 (37.8)	178 (36.2)	155 (38.5)	75 (40.1)	27 (40.3)	1,031 (37.9)
**Hospitals**									
**(C) Transfer**	1 (3.2)	5 (7.2)	21 (6.3)	115^*^ (10.1)	43 (8.7)	37 (9.2)	13 (7.0)	3 (4.5)	238 (8.7)
**(D) Nontransfer**	2 (6.5)	9 (13.0)	21^#^ (6.3)	138^*^ (12.1)	42 (8.5)	36 (8.9)	19 (10.2)	11 (16.4)	278 (10.2)
**Total**	31 (100)	69 (100)	334 (100)	1,139 (100)	492 (100)	403 (100)	187 (100)	67 (100)	2,722 (100)

* : Adjusted standardized residuals in the residual analysis of the chi-square test were ≥1.96 (*p* < 0.05).

# : Adjusted standardized residuals in the residual analysis of the chi-square test were ≤−1.96 (*p* < 0.05).

Chi-square test results revealed significant differences in the distribution of occupational LBP for the number of workers involved in the accident (Table 7). In all groups, occupational LBP occurred most frequently during solo work, accounting for 84.5% of the total cases. Furthermore, occupational LBP occurred more frequently during multi-worker tasks in Groups A and C compared with Groups B and D, whereas it was more common during solo work in Group B.

**Table 7:** Number of workers involved in occupational low back pain incidents.

Number of cases (%)	One worker	Two or more workers	Total
**Social welfare facilities**			
**(A) Transfer**	928^#^ (40.4)	247^*^ (58.4)	1,175 (43.2)
**(B) Nontransfer**	974^*^ (42.4)	57^#^ (13.5)	1,031 (37.9)
**Hospitals**			
**(C) Transfer**	165^#^ (7.2)	73^*^ (17.3)	238 (8.7)
**(D) Nontransfer**	232 (10.1)	46 (10.9)	278 (10.2)
**Total**	2,299 (100)	423 (100)	2,722 (100)

* : Adjusted standardized residuals in the residual analysis of the chi-square test were ≥1.96 (*p* < 0.05).

# : Adjusted standardized residuals in the residual analysis of the chi-square test were ≤−1.96 (*p* < 0.05).

## Discussion

This study investigated the caregiving activities that create a high-risk of occupational LBP in social welfare facilities and hospitals. The findings were divided between assistance activities that involved care recipient/patient transfer and those that did not.

### Transfer assistance

Transfer assistance accounted for approximately half the occupational LBP cases in both social welfare facilities and hospitals. The correspondence analysis indicated that cases caused by transfer assistance in both types of facilities belonged to the same category. These cases mainly occurred during bathing, toileting, eating, and other situations. The residual analysis demonstrated that eating, bathing, and other situations were common in social welfare facilities, while bathing and other situations were common in hospitals. Excluding the other situations, bathing, toileting, and eating situations were therefore considered the caregiving activities most associated with occupational LBP during transfer assistance in social welfare facilities and hospitals.

Few studies have investigated the specific caregiving contexts in which transfer assistance causes staff injury. According to the Ministry of Health, Labour, and Welfare [12], which analyzed the *Reports of Worker Casualties* with four or more absences in 2004, 65.1% of occupational LBP cases in social welfare facilities were caused by transfer assistance. Among these, excluding other situations (44.6%), the most common situations involving transfer assistance were bathing (29.5%), toileting (16.1%), and eating (6.7%). The present study’s social welfare facilities-related findings were consistent with these results.

However, previous studies of workplace injuries in hospitals have categorized all forms of transfer assistance as a single activity, making it unclear which specific transfer-related tasks were associated with occupational LBP. Because hospital patients typically spend more time in bed and require fewer transfers than social welfare facilities care recipients, the incidence of occupational LBP related to transfer assistance in hospitals was lower than in social welfare facilities. However, like social welfare facilities, hospitals also provide transfer assistance for bathing, toileting, and eating to patients with disabilities or injuries. Therefore, although further research is needed to determine the range of transfer-related caregiving activities in hospitals, there seems to be no significant differences in such activities between hospitals and social welfare facilities.

### Nontransfer assistance in social welfare facilities

The correspondence analysis showed a strong association between nontransfer assistance in social welfare facilities and five caregiving situations: sitting, standing, walking, car transportation, and childcare. Moreover, the residual analysis showed that bathing, toileting, diaper changing, lying, sitting, standing, walking, car transportation, and childcare were common. Therefore, nontransfer assistance with bathing, toileting, diaper changing, lying, sitting, standing, walking, transportation, and childcare were considered the caregiving activities most associated with occupational LBP in social welfare facilities.

According to the Ministry of Health, Labour, and Welfare’s analysis report [12], the nontransfer assistance activities most associated with injury, excluding other situations (34.2%), were childcare (20.0%), bathing (18.3%), toileting (10.8%), and diaper changing (10.8%). However, the Ministry did not categorize caregiving activities into lying, sitting, standing, walking, car transportation, and medical support but instead grouped them within the other category. Otherwise, the results of the present study were consistent with those of the Ministry.

Other studies have identified bathing [8,10,13–16], toileting [8,10,13–15,17], diaper changing [10,13,18], lying [8,10], standing [19], and childcare [20–22] assistance as tasks that significantly burden social welfare facilities nurses. However, no studies have indicated that assistance with sitting, walking, or car transportation impose similar strain. Nevertheless, sitting assistance involves lifting a care recipient or adjusting their position to ensure proper posture. Walking assistance here refers to supporting a care recipient while walking and preventing them from falling. Car transportation involves helping care recipients board, disembark, and settle into their seats. These tasks likely place considerable strain on the caregiver’s lower back.

### Nontransfer assistance in hospitals

The correspondence analysis revealed a strong association between nontransfer assistance in hospitals and three caregiving situations: diaper changing, lying, and medical support. Additionally, the residual analysis indicated that, alongside these three situations, sitting was commonly associated with occupational LBP. Therefore, diaper changing, lying, sitting, and medical support can be considered caregiving activities linked to occupational LBP during nontransfer assistance in hospitals.

Because many hospital patients spend a lot of time in bed and cannot move freely, the incidence of occupational LBP related to nontransfer assistance in hospitals was lower than that in social welfare facilities. The Ministry of Health, Labour, and Welfare has not conducted an analysis for hospitals. However, previous studies identified diaper changing [23,24], lying [23–29], and medical support [24,25] as tasks that significantly burden hospital staff. However, no reports have indicated that sitting imposes a similar burden. Nevertheless, due to the aforementioned tasks involved in sitting assistance, it likely places considerable strain on a nurse’s lower back.

### Transfer origin and destination in social welfare facilities

Occupational LBP cases associated with transfer assistance in social welfare facilities mainly occurred during bed-to-wheelchair, wheelchair-to-bed, toilet-to-wheelchair, floor-to-wheelchair, wheelchair-to-toilet, chair-to-wheelchair, and wheelchair-to-chair transfers. According to the Ministry of Health, Labour and Welfare [12], many occupational LBP cases associated with transfer assistance in social welfare facilities were from bed-to-wheelchair (23.7%), wheelchair-to-bed (19.6%), wheelchair-to-wheelchair (8.5%), floor-to-wheelchair (4.9%), and wheelchair-to-toilet transfers (3.6%). However, the Ministry did not include transfers to and from chairs and instead classified these devices as wheelchairs. Considering this difference in classification, this study’s results echoed those of the Ministry.

Other studies have reported that bed-to-wheelchair and wheelchair-to-toilet transfers significantly burden the lower back [10,13]. Meanwhile, there have been no findings indicating that floor-to-wheelchair and chair-to-wheelchair transfers are a significant burden. However, transferring a care recipient from the floor to a wheelchair is most often performed in emergencies when they fall or sit on the floor. This forces caregivers into awkward or high-stress working postures and requires them to lift the care recipient manually. In the present study, transfers between wheelchairs and chairs primarily occurred in bathrooms and communal areas. In bathrooms, frequent transfers occurred between wheelchairs and shower chairs or carriers, while in communal areas, transfers between wheelchairs and sofas were common. The frequency of these transfer tasks may explain the high number of occupational LBP cases.

### Transfer origin and destination in hospitals

Occupational LBP cases associated with hospital transfer assistance mainly occurred during bed-to-wheelchair, wheelchair-to-bed, and bed-to-stretcher transfers. Previous studies have reported that transfers between a bed and a wheelchair and between a bed and a stretcher in hospitals significantly burden the lower back [26]. These transfer tasks may increase the risk of occupational LBP because they often require awkward postures and force nurses to lift patients.

### Priority care activities for improvement

Most occupational LBP cases occurred during transfer assistance, particularly while assisting patients with eating, bathing, and toileting in social welfare facilities and hospitals. The findings of this study reinforce previous studies that identified transfer assistance as a major risk factor for occupational LBP among caregiving and nursing staff [5–7,9–11]. Although transfer-assistive devices have been demonstrated to effectively reduce workers’ physical burden during transfers [30–32], their implementation in Japan remains limited [10,33–35]. In this study, only six LBP cases involved the use of such devices, suggesting that manual transfers remain common and contribute substantially to the high incidence of occupational LBP. Strengthening the use of transfer-assistive devices during activities such as eating, bathing, and toileting is therefore a crucial preventive strategy. Additionally, because many LBP cases occurred during solo transfer assistance, encouraging the collaboration of multiple caregivers may further reduce this risk.

Occupational LBP also occurred during nontransfer assistance activities in social welfare facilities, such as bathing, toileting, childcare, diaper changing, lying, standing, sitting, walking, and car transportation. It was most frequently reported during solo work during day shifts. These findings indicate that preventive measures should not be restricted to transfer assistance. Effective countermeasures could include promoting cooperative work among caregivers, introducing appropriate assistive devices for each type of task, and providing training to improve caregivers’ posture and movements.

In hospitals, occupational LBP occurred primarily during nontransfer assistance tasks, including lying, diaper changing, medical care, and sitting. As in social welfare facilities, it was particularly common among staff working alone during day shifts. To address these risks, preventive strategies such as ensuring adequate staffing, adjusting bed height to an appropriate level, improving working posture, and optimizing the caregiving environment would be beneficial. These findings highlight the need to prioritize preventive strategies targeting high-risk caregiving activities—especially transfer and solo-assistance tasks—through the broader implementation of assistive devices, ergonomic improvements, and cooperative work practices.

This study found that a large proportion of occupational LBP cases occurring during solo work, which is a common scenario in social welfare facilities and hospitals. Performing caregiving assistance alone imposes substantial physical burden on workers, potentially increasing the risk of occupational LBP [19,36]. Therefore, clearly distinguishing between tasks that a single worker can perform and those that should involve two or more workers based on occupational LBP incidence could be an effective prevention strategy.

### Limitations

This study had several limitations beyond data availability. First, the other situations category accounted for approximately 60% of the transfer assistance occurring at social welfare facilities and hospitals. This category included cases in which the activity was either unspecified or did not fall into other specified categories, with many instances of transfer assistance belonging to the former. Second, the transfer origin and destination were not fully recorded in many reports on transfer assistance. A greater variation in results may have been obtained if these details had been more clearly identified. Nevertheless, the findings largely aligned with those of previous studies [12]. Third, 99.4% of the cases involved acute pain, whereas only 0.6% involved chronic pain. This indicates that chronic occupational LBP needs to be considered separately. Finally, the *Reports of Worker Casualties* lacked detailed information on specific tasks and direct pain-related factors, such as pain intensity, duration, and treatment. Although work absence duration was used as a proxy for occupational LBP severity, no significant differences were observed in most of its distributions, and it reflects pain only indirectly. Future studies should therefore incorporate more direct pain-related measures and apply both quantitative and qualitative approaches to evaluate occupational LBP and its associated factors better.

### Strengths

This study discussed occupational LBP cases that are eligible for compensation throughout Japan. In addition, it identified the caregiving activities associated with occupational LBP for each type of facility, including social welfare institutions and hospitals.

## Conclusions

In social welfare facilities and hospitals, caregiving activities linked to occupational LBP commonly included transfer assistance for bathing, toileting, and eating, which were shared risk factors between the facilities. However, the specific nontransfer assistance contributing to occupational LBP varied by setting. Social welfare facilities saw cases involving nontransfer assistance like bathing, toileting, childcare, changing diapers, lying, standing, sitting, walking, and car transportation. In hospitals, nontransfer assistance for lying, diaper changing, medical support, and sitting were identified as contributing to workplace injury. Furthermore, most occupational LBP occurred during day shifts when employees were working alone. Effective occupational LBP prevention strategies should target these high-risk activities according to facility type and the types of caregiving required.

## Supporting information

S1 File Dataset. (XLSX)
